# How Does One “Open” Science? Questions of Value in Biological Research

**DOI:** 10.1177/0162243916672071

**Published:** 2016-10-04

**Authors:** Nadine Levin, Sabina Leonelli

**Affiliations:** 1 UCLA Institute for Society and Genetics, Los Angeles, CA, USA; 2 Exeter Centre for the Study of the Life Sciences (Egenis) & Department of Sociology, Philosophy and Anthropology, University of Exeter, Exeter, UK

**Keywords:** academic disciplines and traditions, archiving and collecting practices, politics, power, governance, methodologies, methods

## Abstract

Open Science policies encourage researchers to disclose a wide range of outputs from their work, thus codifying openness as a specific set of research practices and guidelines that can be interpreted and applied consistently across disciplines and geographical settings. In this paper, we argue that this “one-size-fits-all” view of openness sidesteps key questions about the forms, implications, and goals of openness for research practice. We propose instead to interpret openness as a dynamic and highly situated mode of valuing the research process and its outputs, which encompasses economic as well as scientific, cultural, political, ethical, and social considerations. This interpretation creates a critical space for moving beyond the economic definitions of value embedded in the contemporary biosciences landscape and Open Science policies, and examining the diversity of interests and commitments that affect research practices in the life sciences. To illustrate these claims, we use three case studies that highlight the challenges surrounding decisions about how––and how best––to make things open. These cases, drawn from ethnographic engagement with Open Science debates and semistructured interviews carried out with UK-based biologists and bioinformaticians between 2013 and 2014, show how the enactment of openness reveals judgments about what constitutes a legitimate intellectual contribution, for whom, and with what implications.

## Introduction: Enacting Openness in Scientific Research

The research community has been flooded recently with encouragement to make things “open” in a variety of ways and to a number of audiences. Through an increasing number of formal and informal policies (Research Councils UK 2013b; Nature Publications 2014; White House Office of Science and Technology Policy 2013; World Health Organization 2014), researchers have been urged to disclose a wide range of outputs from their work, ranging from publications to data sets, software, biological materials, and models. Such policies have attempted to codify openness as a specific set of research practices and guidelines that can be interpreted and applied consistently across disciplines and geographical settings. These policies, which are closely tied to the emerging “Open Data,” “Open Software,” and “Open Access” movements (Willinsky 2005), present openness as a way to enhance the transparency, authority, and accountability of research (The Royal Society 2012b; Nature Publications 2014), to promote the accessibility and reusability of research outputs within and beyond the scientific community, and to challenge existing regimes of communication and assessment that are perceived as threatening to the integrity and creativity of researchers (European Commission 2014).

We challenge the assumption that openness is an intrinsically positive goal for science and one that needs to be promoted and rewarded at every step of the research process. We argue that this “one-size-fits-all” view of openness sidesteps key questions about the forms, implications, and goals of openness for everyday research practice. Rather than taking openness as a fixed or singular thing (see Grubb and Easterbrook 2011), we see it as an enactment, a dynamic practice of opening and closing. What researchers choose to open, how, and with whom is a highly situated matter that depends on the goals, preferences, constraints, and institutional settings of the researchers involved. In such decisions, it can be difficult to maintain a clear-cut distinction between public and private spheres or between the various layers of sociality in which research is embedded (Daston 1995). We argue that openness is a *mode of valuing* the research process and its outputs, such that particular forms of work and labor are needed to make things “open” in particular ways. We also take “value” not only to mean economic value in relation to markets but also more broadly to include sociocultural value in relation to communities, and ethical and normative value in relation to societal ideals (Rajan and Leonelli 2013; Birch and Tyfield 2012; Helgesson and Kjellberg 2013; Dussauge, Helgesson, and Lee 2015). This opens up a critical space for moving beyond the economic definitions of value that tend to be embedded in the contemporary biosciences landscape and Open Science policies, and stresses the diversity of interests and commitments that affect research practices in the life sciences.^1^

In the United Kingdom where the research for this paper was carried out, for instance, a particular form of openness has been codified in the UK Government’s “Policy on Open Access” (Research Councils UK 2013b, 1), which focuses on access to journal articles as a first step toward an open scientific culture. In 2013, Research Councils UK (RCUK), the main funder of academic research in the UK, established a policy focusing on the “unrestricted, on-line access to peer-reviewed and published research papers, free of any access charge,” to be realized either through an author-pays model (where authors pay publishers to provide free access to their publications) or through the inclusion of the manuscripts in an open-access repository (see Research Councils UK 2013b on Open Access). It is remarkable that despite its detailed mandate concerning the modes and timing of publication of research papers, the RCUK Policy lacks clarity on how and when other aspects of the research process should be made open, as well as whether and how such openness should be enforced.

The UK Government’s focus on open access of published research outputs is only one of many approaches to openness, and therefore entails moral and evaluative judgments about which types of openness are best for the UK society and economy, and consequently which types of outputs and labor are more valuable than others. Consequently, the RCUK Policy on Open Access poses challenges for researchers because papers contain reference to data sets, software, models, instruments, protocols, and know-how that should also be shared for the contents of papers to be intelligible and reproducible. Recently, an increasing number of funding bodies, learned societies, and journals have begun to promote the inclusion of data and other aspects of the research process within the remit of Open Science (The Royal Society 2012a; Research Councils UK 2013a; The Wellcome Trust 2013; Desjardins-Proulx et al. 2013), but the dissemination of data, biological materials, and methods is for the most part not codified, obligatory, or policed (Schofield et al. 2009). Because papers are the only outcomes that are formally credited and valued by the UK Government, making other components of the research process available is—as we discuss throughout this paper—tremendously challenging. Without formal credit for outputs beyond papers, researchers struggle to negotiate what counts as, and how to be open with, intellectual contributions (Levin et al. 2016).

Consequently, this paper examines how openness is enacted by researchers in their everyday work, as a lens to explore which types of outputs are viewed as valuable or dispensable, which forms of scientific labor are highlighted or obscured, and what consequences this has for knowledge production and professional development. Specific enactments of openness draw attention to specific aspects of the research process: how things are made open or closed reveals judgments about what constitutes a legitimate intellectual contribution, for whom, and with what implications. We argue that the “dilemma of openness” does not only concern whether research results should be made available to the public, or what constitutes useful and useless information (see Grand et al. 2016). It also concerns the shadows of research: the elements and procedures that are taken for granted and little discussed in the planning and evaluation of scientific work, but which unavoidably accompany and support the production of knowledge.

Our framing of openness builds on a body of scholarship in Science and Technology Studies emphasizing that comparisons between “open” and “closed” modes of research are overly simplistic. Openness, as Chris Kelty (2012) emphasizes, is not a natural aspect of science, nor has science ever been fully open or fully closed. In Hilgartner’s (2012b, 267) compelling terms, science entails a “dialectic of revelation and concealment through which knowledge is selectively made available and unavailable,” such that barriers to communication, the closedness of resources, and secrecy are an inherent part of scientific work (Hilgartner 2012a; Balmer 2013; Rappert 2010; Rappert and Balmer 2007). STS scholars have also noted how the modern instantiations of openness that permeate the Open Science movement are intertwined with particular political–economic regimes, such as the increasing commercialization of the biosciences (Birch and Tyfield 2012), and the exercise of proprietary intellectual property regimes like licensing, patents, and trademarks (Calvert 2012). Open Science, with its focus on freedom, democracy, individualism, and free competition, is not necessarily opposed to the proprietary and corporate (Hayden 2010), and can be viewed as a key feature of neoliberal society (Tkacz 2012; Kansa 2014). Openness entails “circuits of exchange” (Lezaun and Montgomery 2015, 4) and, with it, articulations of what is valuable and what relationships exist to generate, ensure, and reinforce such value (Rajan and Leonelli 2013).

Openness, therefore, is not only a technical problem to be solved but is also a social, cultural, and moral issue (Mauthner and Parry 2013; Peters 2014; Nuffield Council on Bioethics 2015). Openness, like “participation” (Tutton and Prainsack 2011; Prainsack 2014), is permeated by notions of social solidarity and altruism, in which the sharing of resources like data is a form of “gift-giving” that entails reciprocal obligations to return the gift (Mauss [1925] 2002; Zeitlyn 2003) through social or economic means (Tschider 2006). Like sharing and donation, openness is predicated on the voluntary labor of researchers and institutions, which often remains unacknowledged and thus undervalued (Mitchell and Waldby 2010; Ankeny and Leonelli 2015). Openness implies uneven social relationships, as both proponents of and participants within regimes of openness are not in neutral positions, but rather are surrounded by epistemic and political constraints and commitments.

Our discussion is based upon in-depth ethnographic engagement in how Open Science policies affect research practices in biology and biomedicine, and particularly experimental work on nonhuman organisms, carried out over the last decade by one of the authors; and the thematic analysis of a specific set of in-depth, semistructured interviews, carried out between September 2013 and January 2014 with twenty-two principal investigators (PIs) working in the fields of systems biology, synthetic biology, and bioinformatics in eleven higher education institutions in the UK.^2^ The interviews aimed to document a wide range of experiences and practices of openness in the life sciences, as well as diverse uses of the notion of value ranging from the purely economic to the societal, scientific, and political. Toward this aim, interviewees were selected on the basis of their active involvement in the Open Science movement (as documented by their participation in Open Science publishing and policy initiatives, and their engagement in related practices). Some researchers were involved through the development of community databases and infrastructures, or the establishment of standards and guidelines. Some encountered Open Science practices through increasingly interdisciplinary, collaborative, or computational work. Others were engaged in a mixture of open and proprietary practices through their involvement with industry-funded research.

In selecting the interviewees, we started from a pool of fifteen subjects whose work was both highly visible as leading the field and strongly associated with Open Science practices. Further subjects were selected through a snowballing method, leading to a sample that includes several practitioners who have worked together on the same projects. This is an unavoidable outcome, given the relatively small size of the community of well-funded PIs in these areas of UK research, and it was useful for our purposes (as will become clear below) since it enabled us to collect and compare multiple perspectives on and responses to the same initiatives and challenges. Because of the highly sensitive nature of the interview materials, in which interviewees often commented on the behavior of their colleagues and institutions, the interviews were conducted under strict confidentiality.^3^

Here we focus our discussion on three case studies that highlight the diverse challenges surrounding decisions about how—and how best—to make things open (see also Nelson 2009). In particular, we discuss three elements that are typically taken for granted by UK-based life scientists as background conditions for their work: the availability of relevant biological materials, computing tools, and freely accessible databases. We found that these elements were brought to the forefront by questions about what openness entails in research practice and how that relates to Open Science policies, with researchers explicitly musing over the value of such resources to themselves and to others. Our cases illustrate how enacting openness:

1. entails decisions about the value of materials to oneself and others,
2. raises questions about attribution and credit within academic research, and
3. requires management, and therefore entails asymmetrical capacities and relationships.

In turn, these issues raise fundamental questions concerning who benefits, and who gets to decide how openness is interpreted and realized; on what timescales, in what locations, and with whom is openness enacted (see Borgman 2012); and how do everyday judgments about openness relate to engrained research practices and existing perceptions of fairness, ownership and intellectual property.

Ultimately, whether openness leads to increased transparency and accountability depends on how, by whom, and for which purposes openness is enacted. As we show throughout this paper, specific instantiations of openness can foster attitudes that many would regard as alien to Open Science mandates, such as a strong sense of ownership of research materials, competition among peers, and closure to sharing outputs and procedures with others (see Evans 2010). We conclude that conceptualizing openness as a performative and valuing process shifts attention away from overly general definitions of Open Science, or from notions that openness is a “magic bullet” to fix society’s problems, and highlights the need to attend to the dynamic and context-dependent considerations involved in opening up particular aspects of research (see Haeussler et al. 2009). Thus, we argue that current scientific and political discussions should focus on what parts of research should be open, how, when, and for which purposes. The variability of situations in which openness is enacted, and the related need to evaluate its implementation on a case-by-case basis, needs to be taken into account by Open Science policies.

## Valuing Biological Materials

Model organisms such as fruit flies, yeast, and mice have long played key roles in the production, replicability, comparability, and integration of results in the life sciences (Ankeny and Leonelli 2011; Leonelli et al. 2013). The ability to control the breeding, modification, and dissemination of these organisms makes them highly valuable to researchers (Clarke and Fujimura 1992; Kohler 1994).^4^ And yet there is little consensus as to how and when specific strains of organisms—and their related data—should be made available as research materials to other laboratories (Rader 2004; Davies 2013; Schofield et al. 2009), particularly when they are essential to research on human disease. In this section, we discuss how researchers negotiate in complex and challenging ways the openness of biological materials such as mice and the ensuing samples, tissues, cell cultures, and data that they generate. By asking about how the openness of experimental organisms should occur, we show how researchers negotiate the value of mice as commodities for future research and as tools for communal work and collaboration.

Our first case involves the generation of transgenic mice containing bacterial artificial chromosomes (BACs), bacterial plasmids containing (often human) genes and promoters, which were generated by a PI in a cell signaling laboratory. Given that the transgenic mice had taken many years and hours of labor to generate, the PI questioned whether and how they should be made available beyond his laboratory. Mice are arguably the most successfully commercialized among the established model organisms, with biomedical researchers typically paying significant amounts of money for access to some of the most popular strains (Huber and Keuck 2013; Davies 2013). The physical setup and expertise of the PI’s group conferred an ability to do research that other people could not do, in his words: “People could make the mice themselves. The point is that where we are with our imaging skills and knowledge, it’s a whole combination of things that puts us in a unique position” (Interviewee 16). For the most part, the PI did not bother with applying proprietary intellectual property regimes like material transfer agreements or patents to his work, because he preferred to control how and with whom he shared physical resources—be they expertise, imaging skills and machines, or the physical BACs and mice. He valued the transgenic mice not only for their use as laboratory animals but also for the range of biological materials that they generated, and for the know-how and labor involved in making them (see related discussions in, e.g., Rheinberger 1997; Hackett 2005).

Indeed, the PI wondered whether there was a “proper” or “right” way that the transgenic mice should be made available, which could fairly account for their value to him as research investments and intellectual property, as well as to their value to the community as reference materials for further experiments. Furthermore, the PI did not know how much information he should provide about his organisms or in what format. The mice were the culmination of many years of work, and the experiments involving them had been varied, resulting in the production of genetic, transcriptomics, and imaging data. Because all of the data were interrelated, the PI was unsure whether to publish a subset of them or release all of them at once. Moreover, he did not know if he should provide raw or annotated data, and if he should put the data in the supplementary information (SI) of journals or field-specific databases—an uncertainty that was shared among many interviewees. This confusion arose not only because of conflicting journal and open science guidelines (see Caulfield, Harmon, and Joly 2012) but also because the PI wanted to make materials available in a way that would allow others to reuse them without compromising his own ability to do original and competitive work in the future.

The PI also questioned *when* to be open in the research process, and particularly whether this should be done before or after publication. Like many other interviewees, he explained how the genomics community had set a precedent for the immediate release of data with the Bermuda Principles^5^ (Maxson, Cook-Deegan, and Ankeny In Preparationg; Cook-Deegan 2007; Strasser 2011), while for other types of data or materials, the time line remained unclear. Many researchers in biomedicine tend to make materials available only postpublication, particularly when the data involve labor-intensive aspects of research. However, the PI wondered if delaying the sharing of data or materials could prove detrimental to the advancement of the research for the wider community. As another interviewee reported, there is often not a clear “time curve” for openness (Interviewee 17), at which researchers could strike a balance between protecting their unique ability to do research, and making a resource available for the greater good of the community.

Lastly, the PI was not sure that making aspects of his research on transgenic mice open would have only beneficial effects. While it may arguably help researchers working in other labs, the PI worried that it may prompt misuse, and have a different—and perhaps detrimental—effect on early career researchers who had been involved in the project. This preoccupation highlights the complexities of enacting openness for results produced by large teams, whose most vulnerable members are precisely those PhD students, postdocs, and technicians most closely involved in the development of materials and the generation of data. The PI worried that freely disseminating mice strains may hurt the publication prospects of early career researchers in his lab, whose chances of retaining a competitive edge over others in the same situation may depend heavily on their exclusive access to specific materials. In other words, mice strains have potentially more value for early career researchers, meaning that openness places different demands on people at different stages of their careers (see Hackett 2005; Pincock 2013).

Such concerns signal the challenges involved in articulating the value of biological materials as commodities for future research, or as tools for communal and collaborative work. The PI acknowledged that making the transgenic mice open would have distinct advantages and disadvantages. On the one hand, making the mice available to the community would enable him to develop research networks, advance his research, and exchange new ideas, as well as enhance his reputation as a provider of replicable research. On the other hand, keeping the mice from dissemination would enable the PI to protect the unique knowledge and resources that he had spent many years developing, and to reap rewards before other research groups could. The mice were produced through many years of unpublished work, and he expected that they would enable him to produce a high-level story for publication in prestigious journals like *Nature* and *Science*, which would undoubtedly help advance his career.

As this case demonstrates, decisions about the openness of materials involve ongoing assessment of value: to individual researchers, to their groups, and to the wider community. Complex challenges are encountered by researchers who conduct labor-intensive research because there is a huge amount at stake in sharing. Being open with research materials brings significant benefits and drawbacks, and so researchers encounter difficulties in negotiating the correct balance—the resources, the timing, the persons involved—between making some aspects of research open and keeping aspects closed (an aspect widely discussed in STS and history of science scholarship, as exemplified by Merton 1973; Latour and Woolgar 1979). This is further enhanced by the considerable worries attached to maintaining material stocks in the long term, given the resources and unclear responsibilities and business models involved in achieving this particularly in the case of mammalian models (Rosenthal and Ashburner 2002). In the case of valuable materials, openness entails a “balancing act between maintaining a competitive edge and…contributing to the community” (Interviewee 22). To deal with this tension, researchers adopt a variety of strategies for enacting openness, ranging from discussions of preliminary experimental data in an informal and verbal capacity with trusted colleagues at conferences (see Wallis, Rolando, and Borgman 2013), to choosing never to disclose data to the wider community, even after publication. These strategies configure the value of research materials in a variety of ways and are shaped by broader norms, policies, and infrastructures, such as the credit and reward mechanisms embedded within contemporary scientific practice, a point to which we turn in the section that follows.

## Valuing Research Tools

Software, with its strong ties to commercial and open source work (Kelty 2008), has come to play an increasingly important role in the organization and interpretation of results in data-intensive research (Stevens 2013). Because software entails authors contributing individual components to a product that is used by a community, it raises questions about who should be legitimately involved versus systematically excluded in research (Kelty 2008; Coleman 2009). The development of software relies on what Shapin calls “invisible labor” (1989), in which value is placed on the collective achievement involved in producing a product, rather than on individual contributions to software development. Given these tensions, software highlights the challenges involved in recognizing, giving attribution to, and legitimizing particular forms of value in academic contexts (see Friend and Norman 2013), in which intellectual outputs need to be formally tracked and assessed at the individual level for career progression and recognition (Kelty 2001). In this section, we discuss how researchers negotiate the openness and value of biological tools like software, drawing attention to the difference between source code and binary formats, and also to the labor involved in generating software. We show how particular forms of attribution and credit play key roles in structuring how software is valued or devalued as a labor-intensive research object.

Our second case involves the development of a software package—which we shall refer to as Software A—for examining cell movement with quantitative imaging data. Like much of the software currently used in computational biology, Software A was made of various machine learning algorithms to analyze experimental data sets of cell images, which enabled users to understand and track cell movement. The PI who had developed Software A made it available at no cost through his website, as long as users registered their information, which enabled the PI to track the number of downloads and the subsequent use of his software. The PI explained that this enabled him to create a user community around the software, in which he could correspond with users and make sure his software was being properly cited and documented. But it also enabled him to make a case for the “impact” of the software by demonstrating that it was being used widely by the research community for a variety of purposes. Thus, the PI enacted the openness of his software in a particular way, such that he could not only track the success of his software but could also ensure that this labor would be valued both by the user community and by the institutions in charge of evaluating the quality of his research.

According to the PI, the development of Software A had been difficult to manage, because it had been developed through a collaborative grant between multiple institutions and PIs. This had created a “delicate situation” when it came to ownership and attribution, because the source code for the software had been created at another institution, and due to changes in research staff, had subsequently been transformed by one of the PI’s postdocs into Software A’s final form. The source code was highly valuable—arguably more so than the software itself—because access to the code made it possible to modify it, reproduce it, and develop it at will. Accordingly, the PI made the decision to make Software A publicly available on his website in its binary format, meaning that users of the software could use its data analysis capabilities, but could not access the underlying source code.^6^ The PI was “not entirely convinced that it is an absolute necessity to release it as a source code” (Interviewee 22), though other PIs emphasized that this was the most valuable aspect of software, which required the most time and effort, and that therefore it was imperative to share it. Making the internal logic of the software available was akin, as another research put it, to “making a laboratory freezer [and every reagent inside it] available” (Interviewee 16). To counter these objections to the decision to withhold the source code, the PI explained that Software A would be released as open source code when the funding for the project was complete, when the publications had been produced, and when the laboratory no longer had the resources to maintain the software. Meanwhile, keeping the software in binary format ensured that “you don’t reveal to your competitors what you are working on” (Interviewee 22).

The PI recounted that he had developed this way of making Software A available on his website in response to earlier negative experiences around intellectual property. On several occasions, he had engaged in collaborations where he had developed software tailored to the needs of specific projects or grants. He explained his frustration at not being included as an author on high-impact papers for which other researchers had used his software to analyze data and generate results. In these cases, he felt that his intellectual contribution and labor had not been given adequate credit. Such concerns pervade academic contexts in which researchers are developing tools and services for the broader community. Biological software development is a relatively new and rapidly evolving field that depends upon a mixture of open source and proprietary applications, and so issues such as attribution and the status of software as an intellectual contribution remain unsettled (see Wiley and Michaels 2004). As another interviewee working on the development of integrated software and database tools for biochemical data commented, “my drive to do everything open, both on the software [and] data side, has certainly in some cases slowed down my career” (Interviewee 3). Other researchers, he explained, tended to look down upon the work of developing tools and services as an inferior form and less valuable form of academic labor.

Such challenges involve choices not only about making research outputs publicly available but also about doing so in formats or at timescales that make them accessible—or inaccessible—in certain ways. In this case, the PI’s strategic decision not to release the software’s underlying code enabled the community to download and use the software free of charge but prevented them from accessing its underlying logic. This particular way of enacting openness reflects specific concerns about the value of academic labor. On the one hand, the PI felt that making the software available as source code would allow the community to use the software in new ways—ways in which he would not be credited as the author—thereby devaluing his creative work. On the other hand, the PI felt that making the software available in binary form would control its distribution and forms of acknowledgment, thereby ensuring that his labor would be credited. In distributing the software in binary form, the PI would make the software of potentially lesser value to the community in that it would limit their ability to reuse the software creatively for other purposes.

Even more so, the case of Software A highlights the challenges of attributing credit (see Acord and Harley 2012) to tools—such as software and algorithms—whose value changes throughout the research process, and may also be perceived differently by contributors with different expertise and interests. Despite the importance of software in the life sciences, many researchers still tend to regard the labor involved in producing software as a service or support activity, which though instrumental to the main goal of producing research claims, does not constitute a research contribution in itself (see Ankeny and Leonelli 2015). This lack of acknowledgment functions as an indirect endorsement of what should—or should not—be recognized as a valuable form of scientific labor, which subsequently affects decisions around hiring, promotion, tenure, and competitive grant processes. In the life sciences, this is compounded because those who develop software are often junior staff who are employed on short-term contracts and frequently shift institutions. Ultimately, this case demonstrates how prevailing mechanisms for allocating credit fail to reward the labor involved in producing software, which affects not only the perception of software as something of lesser value than other outputs such as publications or patents but also the particular way that the openness of software is enacted in academic communities.

## Valuing Communal Resources

Databases play a key role in organizing and making research outputs available to others (Chow-White and Garcia-Sancho 2011). While whole fields of research, such as genomics, have been created around databases like GenBank (Hine 2006; Strasser 2008), databases are also frequently developed and used by more specialized communities (Leonelli 2016). In such situations, databases have difficulty motivating researchers to contribute and curate their data, which require time and effort that receive little attribution and credit (Ankeny and Leonelli 2015). In this section, we discuss how researchers negotiate the openness and value of communal resources such as databases, and the data contained within, drawing attention to the value of raw versus annotated data, as well as the labor required to generate well-curated data in a format—and with adequate metadata—to enable reuse.^7^ We show how the promotion of certain types of openness requires management, and in doing so promotes the asymmetrical value of some things over others.

Our third case concerns the development of a systems biology database that aimed to provide a community repository for the growing interdisciplinary group of researchers who recognized that pooling results could maximize overall knowledge. According to the PI who had developed the database, data-intensive systems biology made heavy use of modeling techniques, and so required access to large volumes of annotated and described data in order to verify the accuracy of results based on data generated from a wide variety of groups and conditions. In data-intensive biology, annotated data were more valuable than raw data, because cleaning, annotating, and formatting made collective results usable across experiments and platforms. As another interviewee said, databases that were “fully curated, where they know what each piece of data means…that’s really valuable. It adds a lot of value to the database” (Interviewee 15).

The PI explained, however, that it was difficult to encourage the users to submit their data to the database because making data available was not a norm in experimental settings in which researchers tended to “cling [to] the data” (Interviewee 22). The availability of annotated data depended on peoples’ willingness to contribute their time and labor to cleaning and uploading data, activities which did not measurably benefit peoples’ careers, as there was “no payback for sharing…you don’t put it on your CV, it doesn’t help your promotion” (Interviewee 2). Obtaining data in a usable form required eliciting the voluntary effort of the research community, and yet did not provide the research community with tangible value, due to a lack of formal rewards for data donation (see Leahey 2008; Edwards et al. 2011). As other interviewees noted, “the value to people at the moment is the publication” (Interviewee 20), such that data donation obligated users to add extra information that did not seem obviously valuable to them, even if it did have value to the wider community.

To encourage users to donate data to the database, the PI gave them access to a series of online data-analysis and visualization tools, which users could employ to make sense of their data after uploading and annotating it. Pairing data submission with data analysis capacities, according to the PI, provided “an immediate return for people” (Interviewee 2) and encouraged them to participate in the database project. The PI relied on the labor of users, and in return provided a reciprocal “gift” to the users with data analysis capacities. Thus, the PI derived value from the users’ data, in that it enabled him to improve his database and algorithms, produce more publications, and—as in the case of Software A—release metrics for “impact” assessments. Similarly, the users derived value from having access to various algorithms for analysis, and by being able to run such algorithms on a large database that went beyond their own data.

The PI acknowledged, however, that he did not provide users with access to the source code or algorithms inherent in the data analysis tools that contained the logic and mathematical models necessary for data analysis, and which could in theory be implemented independently of the database’s java web service. This presented him with a “dilemma of openness” (Interviewee 2), whereby the PI required users to provide annotated data—data that could be reused and reinterpreted—and yet did not provide source code for software tools—ensuring that they could not be reused. The PI required researchers to be open in particular ways with their data, but yet was not open with all of the components of the database. Acknowledging the dilemma of openness, the PI explained that his actions were motivated by a fear that people would not donate their data to the database unless they were provided with some incentive. He said: “our experience with [the database] was that very few people actually use[d] it for data sharing until we made it possible for them to analyze their data online” (Interviewee 2). The PI feared that if the underlying source code was made available to people, they would “use the web service code locally to do their own analysis, and we would never see the data” (Interviewee 2).

This case shows not only how there are multiple ways of valuing data—as objects that can be disseminated on their own, or as objects that require annotation and metadata (see Leonelli 2016)—but also that particular ways of enacting openness confer value to some things and strip value from others. By requiring users to donate annotated data to the database, the PI valued the reuse of data by the wider community and devalued the time-intensive labor required to annotate and donate data in a way that made it reusable. However, this case also shows how data donation becomes normalized—by, for example, integrating it into data analysis capabilities—such that it becomes as commonplace as writing in a laboratory notebook. The transformation of data donation into something routine and required, however, does not necessarily reward or recognize the forms of labor implicit in the cleaning and annotation of donated data. Because openness is enacted in a variety of ways in practice, promoting one version of openness requires management and work, which legitimizes some scientific outputs and practices and delegitimizes other.

Ultimately, particular enactments of openness lead to asymmetrical capacities and valuations among the people involved, leading to tensions between those who make decisions around what should be open and those who are meant to use open resources. As this case highlights, the openness of communal resources entails asymmetrical capacities of database users and managers, as those in control of data demand and place value on the openness of some things—in this case, annotated data—while promoting the closure of other things—in this case, the logic of data analysis. At stake here is who has the authority to decide how openness should occur, what aspects of research should remain open and closed, and whether this affects the research capacities of the different actors involved. These tensions and challenges pertain not only to specific research communities, such as the one discussed in this case, but also to broader government-wide policies on Open Science, a point to which we turn in the concluding section.

## Conclusions: The Dilemma of Openness between Policy and the Bench

In the past decade, the Open Science movement has emerged as a champion of scientific progress, emphasizing its ability to foster transparency, equality, and innovation through openness. This paper critically examined not only how openness is negotiated by researchers but also how politics enters the negotiation. Because openness must be accomplished rather than being automatically secured, this examination highlights how particular work is required to make certain things open in certain ways and to certain people. Openness—whether it involves disclosure, dissemination, sharing, or reuse—comes in degrees and varieties. Like shadows, openness is the result of nuanced encounters between light and darkness, whose visible results reflect both the obstructions and specificities of each setting. Again like shadows, openness is inherently positional and relational and is subject to dramatic qualitative shifts depending on the characteristics of the locations involved or the personal relationships between the individuals and groups involved. Whether this is explicitly acknowledged or not, openness entails judgments about what counts as a valuable research output or practice, such that particular enactments of openness lead to the endorsement of some things as valuable, and others as not. It is not just a question of what should be made open but also about how particular instantiations of openness value some forms of care and labor over others.

Taken together, these cases show how openness, as a process and practice, is not in itself positive or negative, but rather its role and implications constantly shift across institutional settings and research networks, and in relation to given resources and priorities. Examining openness as a mode of valuation becomes increasingly important in the context of Open Science policies, where particular forms of openness are frozen and embedded in specific social norms, economic structures, and political reasoning. When openness is codified in policy, it not only enacts particular things as open and closed but also performs certain values, such as defining some research outputs and practices as more valuable than others. Although Open Science policies benefit society in numerous ways, they also carry assumptions about what, who, when, and how openness should occur (Whyte and Pryor 2011). These policies promote normative understandings of the economic and sociocultural significance of the processes and products of research whereby value is often stripped from outputs like data, software, and databases, leading these entities to remain in the shadows, unacknowledged (or, in the case of data, acknowledged in ways that obscure the labor and care necessary to effectively disseminate these outputs as valuable in and of themselves, rather than as evidential props for claims made in journal publications).

Ultimately, there are profound tensions and difficulties in formalizing this diversity of modes, circumstances, and outcomes of openness and valuing processes. We have shown that those situations in which some activities and outputs are valued over others, including situations where researchers disagree on what is or is not valuable, are also sites at which openness is most controversial. The overly general approaches to Open Science typified by the RCUK Policy on Open Access create confusion among researchers, because they do not leave space for the specific contexts and individual needs of everyday research. They do not provide researchers with parameters to decide, for example, when data should be placed in pdfs within a journal’s SI or in annotated repositories, or if data should be released immediately in an uncurated form or several months after publication in a more polished form. Sites where researchers experience the “dilemma of openness” are also those sites where Open Science policies can be interpreted in a variety of different ways, or perceived as fruitful, pointless, or threatening by researchers, depending on their circumstances. This situation encourages researchers to view strategies for Open Science as risky and unrewarding, thus creating feelings of ambivalence that may well encourage disengagement and anomie (Merton 1963; Hackett 2005). Given the diverse instantiations of openness, and the challenges that can come with providing too narrow a definition of openness in official policies, we strongly support the evaluation and enforcement of Open Science guidelines on a case-by-case basis.

In conclusion, we have found that many researchers resist the “imperative to share” (Lezaun and Montgomery 2015) those things that are most valuable to them, feeling that it compromises the integrity or future capacity of their research. In those cases, value lies not in what is shared, but rather in what is *not* shared: in those things that may not be made tangible, visible, and countable by Open Science policies. As such, the processes of valuation espoused by Open Science policies are, at times, in tension with those practiced by researchers, as researchers involved in more “invisible” forms of labor, like software development and data curation (Howe et al. 2008), have little incentive to be open in ways that governments or communities may expect. For researchers, value often lies not in the final products or commodities but rather in the labor-intensive processes required to collate and disseminate research, which entail skill and know-how. The openness of these things, which leave traces in the final outputs of research, require constant negotiation and flexibility to adapt to changing research conditions. As one researcher emphasized during an interview, he was sometimes more reluctant to be open with papers than with patents or data, because it was “very difficult not to give away know-how in a paper, [and] if it’s a paper about a process, that’s the problem” (Interviewee 8).

Such questions about value also speak to broader concerns about how data and other research-related objects are being made into commodities––a process that underpins and motivates Open Science policies at least as much as the wish to enhance the excellence and impact of scientific research, as well as social engagement in its processes and outcomes (Leonelli 2016). This risks making researchers into “interchangeable data collectors” and obscuring the uneven politics and power relations that practices of dissemination and sharing entail (Mauthner and Parry 2013). Because people have close and ever-changing relationships with the objects of their labor, the commodification of research processes and outputs is inevitably in tension with the entangled and relational nature of everyday research. When things become open in the ways mandated through some of the existing Open Science policies, they risk losing their ties to specific individuals and contexts, such that “openness” ceases to be governed by localized principles of trust and gifting, and instead is governed through generalized principles of “economic value.” Once things begin to circulate, researchers lose control over their products and labor, such that their work may not be used in their interest, or such that it may be used in ways that do not line up with their own values. In other words, the forms of openness promoted by governments and research communities risk “making up” research subjects (Tutton and Prainsack 2011) in normative ways that restrict the circulation of knowledge and resources. Ultimately, openness does not serve all equally (Kansa 2014; Bezuidenhout et al. 2016), raising questions about what openness makes visible and invisible.

## Notes

1. As Rob Kitchin has noted: “Much more critical attention needs to be paid to how open data projects are developing as complex sociotechnical systems…to date, efforts have concentrated on the political and technical work of establishing open data projects, and not enough on studying these discursive and material moves and their consequences” (Kitchin 2013, 66).

2. This paper focuses on specific aspects of the interviews that were of interest to the authors, and which corroborated their existing ethnographic expertise in experimental practices in biology. A full thematic analysis of the interviews, detailing their overall content and sketching recommendations as a result, is currently under review (Levin et al. 2016).

3. All interviewees signed a consent form detailing the ways in which data would be handled by the research team. Ethical procedures were approved by the Ethics Committee of the College of Social Science and International Studies at the University of Exeter.

4. There is ample scholarship, particularly within the history of science, on the significance and uses of model organisms in biological research, much of which is reviewed in Ankeny and Leonelli (2011). Particularly notable for our purposes is Robert Kohler’s detailed study of the research practices and modes of dissemination construed around the fruit fly *Drosophila melanogaster* by members of the Morgan laboratory in Columbia in the 1920s, which evidences the care and effort necessary to select, develop, and use nonhuman models as a common research tool and reference point for a numerous and geographically fragmented research community. On the significance of developing model organisms and related infrastructures and norms as means to generate scientific communities in the first place, see Leonelli and Ankeny (2015).

5. The Bermuda Principles were established in 1996 at a summit in Bermuda, during the early stages of the Human Genome Project, to outline the rapid and public release of DNA. The scientific community agreed that all DNA sequence data should be released to publicly accessible databases within twenty-four hours of its generation (Human Genome Organisation [HUGO] 1996).

6. Here, we reference the distinction between “source code” and “binary format.” Source code refers to the software program’s line-by-line text, which has been written by programmers in a particular language, giving programmers the ability to change and modify the text. Binary format refers to source code that has been transformed into a set of instructions that a given computer or operating system understands how to run in order to make the software work, but which does not give access to line-by-line text.

7. Databases are arguably a specific type of software, and thus many of the observations made in the previous section apply to this case, too. At the same time, we wish to consider databases separately because of the different type of labor, user consultations and resourcing (data donation and reuse strategies) associated with setting up and maintaining them, of which the development of adequate software is only one component.
